# T Cell Hierarchy in the Pathogenesis of Psoriasis and Associated Cardiovascular Comorbidities

**DOI:** 10.3389/fimmu.2018.01390

**Published:** 2018-06-19

**Authors:** Fabio Casciano, Paolo D. Pigatto, Paola Secchiero, Roberto Gambari, Eva Reali

**Affiliations:** ^1^Department of Morphology, Surgery and Experimental Medicine and LTTA Centre, University of Ferrara, Ferrara, Italy; ^2^Department of Dermatology and Venereology, I.R.C.C.S. Istituto Ortopedico Galeazzi, University of Milan, Milan, Italy; ^3^Department of Life Sciences and Biotechnology, University of Ferrara, Ferrara, Italy; ^4^Laboratory of Translational Immunology, I.R.C.C.S. Istituto Ortopedico Galeazzi, Milan, Italy

**Keywords:** psoriasis, skin, inflammation, psoriatic arthritis, TCR repertoire, comorbidities

## Abstract

The key role of T cells in the pathogenesis of cutaneous psoriasis has been well described in the last decade and the knowledge of the relative role of the different subsets of T cells in psoriasis pathogenesis has considerably evolved. Now, it is clear that IL-17A-producing T cells, including Th17/Tc17, have a central role in the pathogenesis of cutaneous psoriasis and therapies blocking the IL-17A pathway show high clinical efficacy. By contrast, the contribution of IFNγ-producing T cells has progressively become less clear because of the lack of efficacy of anti-IFNγ antibodies in clinical studies. In parallel, the role of CD8^+^ T cells specific for self-antigens has been revived and increasing evidence now indicates that in psoriatic skin the majority CD8^+^ T cells are present in the form of epidermal tissue-resident memory T cells. In the last years it also emerged the possibility of a contribution of T cell recirculation in the pathogenesis of psoriasis and its systemic manifestations. The aim of this review is to define a hierarchy for the different subsets of T cells in the T cell-mediated inflammatory cascade in psoriatic skin. This analysis will possibly help to distinguish the subsets that initiate the disease, those involved in the establishment of the self-sustaining amplification loop that leads to the cutaneous clinical manifestations and finally the subsets that act as downstream players in established lesions. Specific T cell subpopulations finally will be considered for their possible role in propagating inflammation at distant sites and for representing a link with systemic inflammation and cardiovascular comorbidities.

## Introduction

Psoriasis is a chronically relapsing inflammatory disease of the skin affecting about 2% of the population worldwide.

Histologically, psoriasis is characterized by three principal features: epidermal hyperplasia, leukocyte infiltrate, and an increased number of tortuous and leaky vessels in the dermis (1–3). In recent studies, the presence of lymphoid aggregates/memory T cell clusters in the dermis of psoriatic plaques has been reported (4, 5).

20–30% of patients with psoriasis also develops psoriatic arthritis (PsA) and there is evidence that psoriasis is associated with systemic inflammation and with comorbidities, such as cardiovascular disease (6–12).

With regards to the pathogenesis, it is now emerging that psoriasis is an immune-mediated disease with a central autoimmune component mediated by T cells. Specifically, the disease pathogenesis involves a dynamic interplay between dermal dendritic cells, T cells (CD8^+^ autoreactive T cells, Th1, and Th17) and keratinocytes giving rise to a self-sustaining inflammatory cycle that develops around the TNF/IL-23/IL-17A axis (13–17). Despite this evidence, the hierarchical sequence of T cell-mediated events in the psoriatic inflammatory cascade is not completely defined. On the basis of the current literature and results of cytokine-blocking therapies it is possible to distinguish, in the T cell-mediated psoriatic inflammatory cascade, (i) an initial skin T cell activation phase, (ii) the establishment of chronic inflammation, (iii) the maintenance of clinically established lesions, and (iv) the egress from the skin of specific subsets of T cells that could possibly take part in the development of extra-cutaneous manifestations of psoriasis, including joint inflammation and cardiovascular comorbidities (18, 19).

## T Cell Hierarchy in the Formation of Psoriatic Plaques

T cell responses against self-antigens in psoriasis are initiated by dendritic cells in the dermis of pre-psoriatic skin (20). Mature dermal dendritic cells can produce TNFα and IL-23, present self-antigens, and stimulate the activation of autoreactive CD8^+^ T cells together with a fraction of CD4^+^ T cells polarized toward Th17 phenotype or the IL-17^+^IFNγ^+^ pathogenic Th1/Th17 as described by Annunziato and co-workers and Eisdmo and co-workers (20–23).

**Figure 1:**
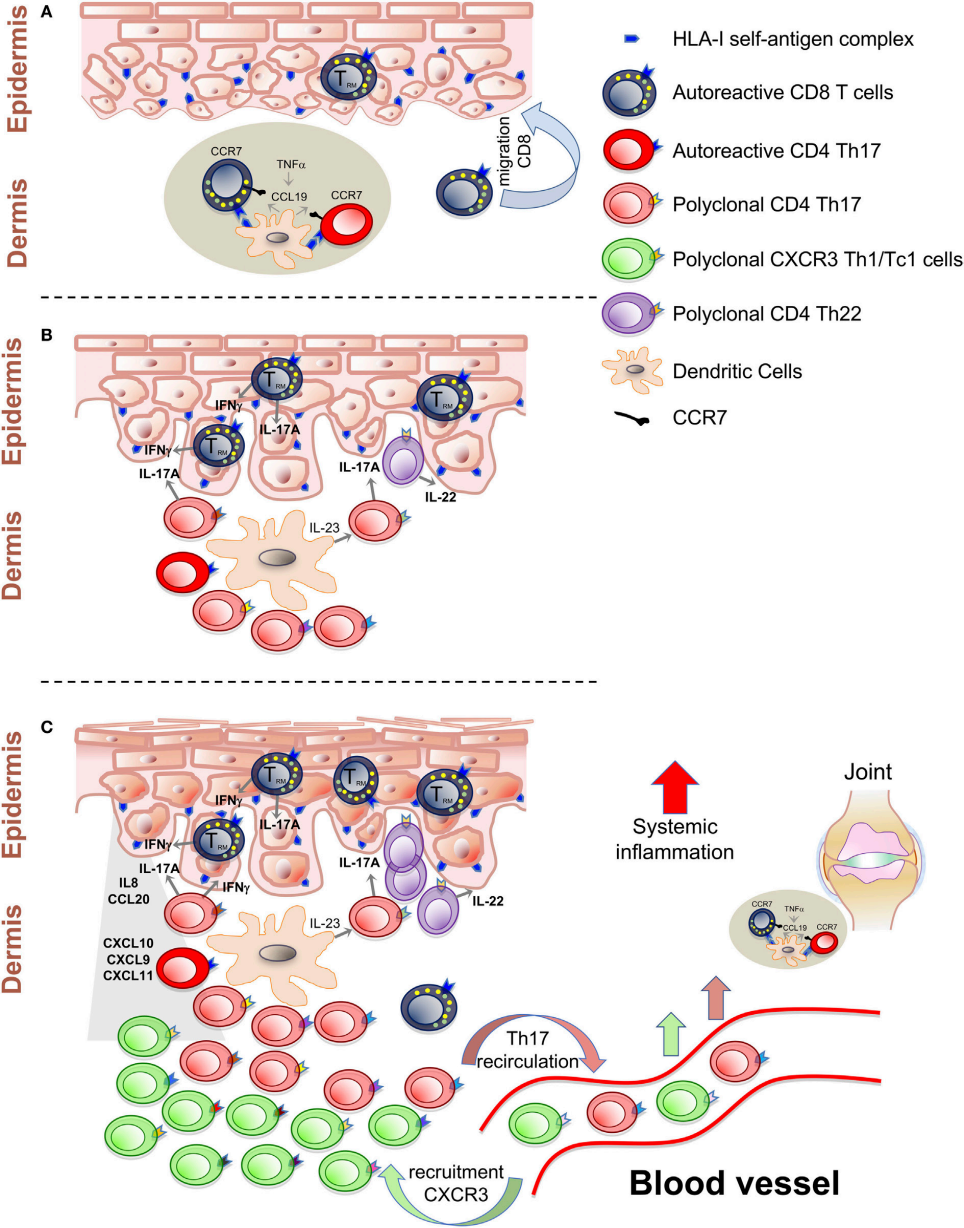
T cell-mediated events in the psoriatic inflammatory cascade. **(A)** Activation of autoreactive T cells by self-antigens presented in the dermal lymphoid aggregates. Establishment of CD8^+^ T_RM_ cells as central autoimmune component of disease pathogenesis and potential mechanisms of site-specific disease memory. **(B)** Polyclonal T cells proliferation and Th17/Tc17-mediated inflammation around the IL-23/IL-17A axis. **(C)** Recruitment of Th1/Tc1 cells, with multiple specificities, from the blood stream, induced by CXCL10 chemokines. Recirculation of T cells from the skin to the blood can spread inflammation at systemic level and at distant sites.

Activated T cells can migrate to the epidermis and recognize epidermal autoantigens and possibly progress toward differentiation to tissue-resident memory T cells (T_RM_) characterized by CD69^+^CD103^+^CCR7^−^CD45RA^−^CD62L^−^ phenotype (24, 25). Recognition of epidermal autoantigens by Tc1/Tc17 induces the secretion of cytokines, including IL-22, that mediate the initial phase of epidermal hyperproliferation and activation of keratinocytes (Figure 1A). These will produce chemokines and antimicrobial peptides, which lead to the progression of the inflammatory process (26–29).

Epidermal autoantigens include LL-37 antimicrobial peptide expressed by keratinocytes, keratin 17, and melanocyte-derived antigen ADAMTS-like protein 5 (ADAMTSL5) recognized by IL-17A-producing CD8^+^ T cells, restricted by HLA-C*06:02 (26, 30–35).

This evidence together with early studies strongly supports that CD8^+^ T cells represent the autoimmune core of the disease. In psoriatic skin lesions, CD8^+^ T cells accumulate in the form of T_RM_ with a pathogenic IFNγ–IL-17A cytokine profile that were detected also in resolved psoriatic lesions and identified as potential mechanisms of site-specific disease memory (20, 25, 36–42).

The major role of CD8^+^ T cells is also strongly supported by the fact that the main psoriasis risk gene is the HLA-class I allele HLA-C*06:02 on psoriasis susceptibility locus 1 on chomosome 6 and additional HLA-class I alleles are associated with psoriasis (43, 44).

Among tissue-resident T cells in human psoriatic skin, Clark and co-workers have recently shown that the vast majority has an αβ TCR (42), despite γδ T cells have been reported to play a role in the production of IL-17A and maintenance of inflammation (45, 46).

As regards to the TCR repertoire, studies of TCRBV chain sequencing showed mono or oligoclonal expansions of T cells mainly of CD8^+^ lineage in psoriatic skin (47–49). TCRBV expansion indicates antigen-driven CD8^+^ T cell responses, whereas the CD4^+^ T cell fraction was reported to be polyclonal (50). In patients with cutaneous psoriasis, however, the presence of oligoclonal TCRBV expansion in peripheral blood is debated (51, 52). Two different studies of high-throughput sequencing of the entire TCR repertoire have shown that alongside with a limited oligoclonally expanded T cell subpopulation in psoriatic skin that was in part retained in resolved lesions, there was a vast majority of polyclonal T cells (42, 53). This data suggests that similarly to what has been described in PsA by Winchester and co-workers, antigen-driven mono and oligoclonally expanded T cell populations represent a limited component of the T cell reactions in psoriatic skin, whereas the vast majority is represented by polyclonal CD4^+^ T cells that are not present in resolved lesions (54).

This evidence together confers to autoreactive CD8^+^ T cells a dominant role in the plaque formation and in psoriasis recurrence and possibly a common role in psoriasis and PsA. It also underlines the importance of mechanisms of T cell plasticity and polarization toward pathogenic phenotypes.

With respect to CD8 T cell targetability, a recent study on a AGR mouse model of psoriasis has reported the accumulation of epidermal CD8^+^ T cells during psoriasis development which was associated with IL-17A production and increased keratinocyte proliferation. Importantly, in this study injection of anti-CD8 antibody completely prevented psoriasis development (29).

Nevertheless, it is likely that Tc1/Tc17 autoreactive CD8^+^ T_RM_ cells overlap their features and functions with the cells that under physiological conditions work as immune sentinels at barrier tissue. For this reason in a clinical setting, it could be difficult to target epidermal CD8^+^ T_RM_ cells by the available immunotherapeutic tools without incurring in major adverse effects (20, 28, 29, 37).

As regards T helper cells, they have been reported to be more abundant in psoriatic skin lesions. While initially they were described mainly as Th1 cells, more recently the attention has been focused on IL-17-producing cells and the IL-23/Th17 axis clearly emerged as central in the control of the pro-inflammatory cycle in psoriatic plaques (14, 15). The high efficacy and fast outcome of IL-17A blocking therapies in resolving cutaneous clinical symptoms has strongly supported this concept (28, 55, 56). In addition, at skin level an expanded subset of T cells that produces IL-22 but not IL-17A (Th22) with a main role in the induction of acanthosis was identified. Importantly, these cells were found to be present also in resolved psoriatic lesions (57, 58).

In line with the key role of IL-17A, the main gene variants associated with psoriasis outside the MHC locus are single nucleotide polymorphisms, belonging to the IL-23/IL-17A axis (*IL23R, IL12B*, and *IL23A*) and the NF-κB pathway (*TNFAIP3*) (44, 59). It is, however, possible to hypothesize that Th17 or pathogenic Th1/Th17 cells could be expanded in the dermis as an event downstream of the epidermal autoimmune T_RM_ and occurs through bystander activation mechanisms like the one described by Winchester and co-workers in psoriatic synovial tissues (54).

In this view, the cycle that develops around the IL-23/IL-17A axis could involve some antigen-driven populations and a considerable fraction of polyclonally expanded T cells that can represent the second step of the pro-inflammatory cascade, responsible for the amplification of inflammation and for the exacerbation of clinical symptoms (Figure 1B).

As a consequence of the strong evidence of the central role of IL-17A-producing cells, the relevance of Th1 cells and IFNγ has become less clear. In lesional skin of psoriasis patients, Th1 cells and IFNγ levels are clearly increased (60, 61). However, in a small pilot study in patients with psoriasis, treatment with a humanized anti-IFNγ antibody induced improvement of histological and some clinical parameters but only minor therapeutic effects (62).

These controversial findings focus on the complexity of the interplay between the Th1/Tc1 and Th17/Tc17 cells in the pathogenesis of psoriasis and it is not clear how the marked increase of IFNγ and IFNγ-producing cells in psoriatic skin can actually link with the failure of IFNγ-blockade to show therapeutic efficacy.

Results from a study by Kryczek et al. have suggested that IFNγ exerts one of its effects by programming myeloid dendritic cells to produce CCL20, ligand of CCR6, and to secrete IL-23. This in turn would favor the recruitment and expansion of IL-17A-producing cells (63, 64). On the other hand, IFNγ mRNA is markedly upregulated in psoriatic plaques and noticeably IFNγ-induced genes, such as *CXCL9, CXCL10*, and *CXCL11* are strongly increased in psoriatic lesions (65).

In line with this evidence, we have previously reported gene expression data in psoriatic skin showing significant enhancement of *CXCR3* and *CXCL10* expression with an inverse correlation between the circulating fraction of CXCR3^+^ CD4^+^ effector memory T cells and the severity of cutaneous psoriasis (66, 67). Therefore, we can postulate an ultimate downstream phase in the psoriatic cascade, driven by the CXCL10/CXCR3 axis which induces the recruitment of Th1/Tc1 from the blood stream (Figure 1C).

## T Cells in the Pathogenesis of PsA

Psoriatic arthritis develops in a fraction of patients with psoriasis and in the majority of cases it follows the development of the cutaneous disease by a mean of 10 years (68).

In addition to the skin, PsA targets the attachment sites of ligament to bone (entheses), the peripheral joints, and the spine (12, 69).

Enthesitis is indeed a distinctive feature of PsA and it has been hypothesized that, in PsA joints, inflammation can start from the entheses. The disease progression, in patients with PsA, can finally lead to destructive bone loss and 67% of patients exhibit signs of erosive bone disease (70).

Similarly to psoriasis, T cells are involved in the pathogenesis of PsA and reduction of CD3^+^ cells in PsA synovium correlated with the clinical response to biological therapies (71).

In PsA patients, Canete and co-workers have evidenced the presence of lymphoid aggregates in synovial tissues that was significantly reduced by TNF-blocking agents. This result could be paralleled by the observation of lymphoid aggregates in psoriatic skin and the role of CCR7/CCL19 axis, modulated by TNF in the initial clustering of dendritic cells and T cells in the dermis (4, 5, 72–75).

In line with the concept of shared pathogenic mechanisms between psoriasis and PsA, Belasco and colleagues provided the evidence that gene expression in PsA synovium was more closely related to gene expression in the PsA patient skin than to gene expression in the synovium of patients with other forms of arthritis. *IL17* gene, however, was upregulated more in skin than in the synovium, whereas *TNF* and *IFN*γ were similarly upregulated in both tissues (76).

As regards the TCR repertoire analysis in PsA joints, Tassiulas et al. (47) showed oligoclonal and monoclonal T cell expansions in the synovial tissue, some of which were shared with the skin. In a subsequent study, Curran et al. performed TCR β-chain nucleotide sequencing in peripheral blood and synovial tissues/fluid showing that 76% of the clones in inflamed tissues were polyclonal, whereas 12% of the expanded clones had structurally homologous CDR3 β-chain sequence and were only CD8^+^ in lineage (54). Interestingly, some of the expanded CD8^+^ clones identified in the synovial tissue were present also in peripheral blood and joint fluid.

A second population of moderately expanded inflammation-related clones, that were either of CD4^+^ or CD8^+^ lineage, was identified only in inflamed synovial tissue and joint fluid. These cells could represent a secondary consequence of the inflammation induced by other proliferating clones.

Finally, one major population consisted of unexpanded polyclonal CD4^+^ T cells that did not persist in the tissue during methotrexate treatment, were most likely effector memory CD4^+^ T cells recruited by inflammatory chemokines released by other cell populations. The lack of clonal expansion in CD4^+^ T cells suggests a lower hierarchical role in driving inflammation.

These findings together suggest a role for cognate T cell responses in the pathogenesis of PsA and further suggest that T cell clones specific for identical or homologous antigens in skin and synovium may represent central elements in promoting inflammation in both tissues (47).

It remains to be established how self-reactive T cell responses in the skin, can be mechanistically linked to the one found in the synovial fluid and how these events can occur with years of time-lapse.

To this end, genetic association studies can provide some interpretation keys. In addition to the HLA-C*06:02 which is common with psoriatic plaque, additional HLA-alleles are associated with PsA. These HLA-alleles include B*08:01:01, B*27:05:02, B*38:01:01, and B*39:01:01 (77).

This underlines the importance of CD8^+^ T cells recognizing HLA-class I associated self-antigens in the pathogenesis of PsA and suggests that the autoimmune basis of PsA may be even broader than the one of cutaneous psoriasis. In this view, enthesis that anatomically links mechanical stress to immunologically active tissue (synovium), could be central for the pathogenesis (78).

Entheses are commonly subject to microdamage associated with local cytokine release, which may evolve into subsequent inflammation (79, 80). Inflammation in turn can favor cross-presentation of self-antigens.

It is, therefore, possible that recirculation of pathogenic skin T cells recognizing common self-antigens can start subclinical inflammation by localizing to the synovioenthesial complex (Figure 1C).

Patients with psoriatic disease have shown an increased level of circulating CCR6^+^ CD4^+^ T_EM_ and T_EFF_ cells that correlated positively with systemic inflammation (66). Therefore, it is possible to hypothesize that a fraction of IL-17-producing T cells recirculates from the skin and relocalize to the entheses.

This is reinforced by the findings of Sherlock et al. reporting that IL-17A^+^ cells locate mainly at the entheses and synovial tissues and by the evidence that among the genetic risk factors outside the MHC locus there are gene variants falling into the IL-23/IL-17A axis (77, 80–85). Importantly, it has been reported that IL-23 is expressed in synovial membrane with ectopic lymphoid tissue (78).

The fraction of IL-17A-producing cells recirculating from the skin could, therefore, determines subclinical inflammation and favor, in the long-term, cross-presentation of self-antigens and epitope spreading phenomena (73, 78).

In established PsA, it has also been reported an increased percentage of IL-17A-producing CD8^+^ T cells in synovial fluid which correlate with bone erosion and disease severity (86–88).

In the light of a hierarchical T cell clonality in psoriatic tissue and the evidence of the multiple-specific clones, including EBV-specific clones, observed in synovial fluid, we can postulate that massive recruitment of T cells with Th1/Tc1 phenotype from peripheral blood occurs as a consequences of inflammatory chemokine release (54). By analogy with the observation in psoriatic skin and by evidence from the data provided by Gladman and co-workers it is possible that CXCL10/CXCR3 axis can act as a downstream cell recruitment mechanism of tissue inflammation common to cutaneous psoriasis and PsA (89–91).

## T Cells in Psoriasis-Associated Cardiovascular Comorbidities

Increasing epidemiological and clinical evidence indicates that psoriasis is an independent risk factor for cardiovascular disease (92, 93).

As a consequence of poorly controlled tissue inflammation, psoriasis patients can develop systemic inflammation and atherosclerosis. In this process, it has been postulated that inflamed tissue-derived factors or cells may directly affect distant vessels for the development of athero-thrombosis (7, 94, 95). Nevertheless, the cellular mechanisms that link the cutaneous manifestations of plaque psoriasis with the initiation and progression of atherosclerosis in psoriasis patients are largely unknown.

A recent study on human tissues showed that psoriasis and atherosclerosis exhibit significant overlap of their transcriptomes and in particular that *TNF, IFNg*, and IFNγ-induced genes, which are common between psoriasis and atherosclerosis may provide the link between the two diseases (96). By contrast, *IL-17A* and *CCL20* were higher in psoriasis than in atherosclerotic tissue, whereas *IL17R* gene was expressed at similar levels.

Because of the link between IL-17A and neutrophil infiltration in atherosclerotic plaques and its key role in the pathogenesis of psoriasis it has been suggested that the IL-17A/neutrophil axis could take part to atherogenesis associated with psoriatic disease (97). Nevertheless, the role of IL-17A in psoriasis-associated atherosclerosis is still controversial. Indeed, Usui et al. reported that IL-17A deficiency protected against atherosclerosis in apoE^−/−^ mice due to reduced macrophage infiltration and inflammatory cytokine secretion in the lesions (98).

Other mouse studies have indicated that IL-17A may promote plaque stability by contributing to fibrous cap formation (99). Collectively, the results indicate that IL-17A may exert both anti- and pro-atherogenic effects, depending on the inflammatory context. However, further studies will be necessary to clarify the contribution of T cells recirculating from the psoriatic plaque in the development of atherosclerosis.

## Implications for the Development of Therapeutic Protocols

From this analysis it emerges a differential contribution of the individual subsets of T cells in the pathogenesis of psoriasis, PsA, and associated cardiovascular comorbidities namely, atherosclerosis. In particular, TNFα has a relevant role in inducing the CCL19/CCR7-mediated formation of clusters of dendritic cells and T cells in both psoriasis and PsA. It also emphasizes the role of IL-23/IL-17 axis in the amplification loop critical for the clinical manifestations of cutaneous psoriasis and possibly in the initial phase of join inflammation.

On the other hand, the possibility of a third step of CXCL10/CXCR3-mediated recruitment of Th1/Tc1 cells from the blood stream may explain the apparent controversy between the high amount of IFNγ-producing cells and the low therapeutic efficacy of anti-IFNγ antibody treatment.

## Conclusion

By defining the hierarchy of the T cell-mediated events of the psoriatic cascade in psoriatic plaques it is possible to distinguish a core antigen-driven oligo or monoclonally expanded autoreactive CD8^+^ T cell component, a secondary self-reactive CD4^+^ T cell component, a polyclonal CD4^+^ Th17 and pathogenic Th1/Th17 population amplified by the IL-23/IL-17A axis, and a downstream recruitment of CXCR3^+^ T cells with different specificities induced by the upregulation of CXCL10 chemokine. Similarly in PsA the analysis of the TCR repertoire has evidenced central antigen-driven expansion of mainly CD8^+^ T cells and broad polyclonal CD4^+^ T cells expansion. In addition, a group of common clones were also expanded in peripheral blood of patients with PsA and T cell clones specific for identical or homologous antigens were present in both skin and in synovial tissues. These may represent central elements in promoting inflammation in both tissues. In addition, oligoclonally expanded clones in peripheral blood of patients with PsA may also suggest that T cell recirculation can represent a mechanistic link between skin and joints inflammation.

## Author Contributions

FC wrote parts of the manuscript and prepared the figures. PP collaborated to the writing. PS and RG discussed the content and critically revised the manuscript. ER supervised the work and wrote the final version of the manuscript.

## Conflict of Interest Statement

The authors declare that the research was conducted in the absence of any commercial or financial relationships that could be construed as a potential conflict of interest.
